# A critical exploration of stigma interventions and programs for queer peoples across the life course in Canada: a scoping review protocol

**DOI:** 10.1186/s13643-025-02917-w

**Published:** 2025-10-24

**Authors:** Jad Sinno, Judith Logan, Jesse Carliner, Amaya Perez-Brumer, Carmen Logie, Daniel Grace

**Affiliations:** 1 Dalla Lana School of Public Health, University of Toronto, Toronto, ON Canada; 2 University of Toronto Libraries, University of Toronto, Toronto, ON Canada; 3 Factor-Inwentash Faculty of Social Work, University of Toronto, Toronto, ON Canada; 4 United Nations, University Institute for Water, Environment, & Health, Richmond Hill, Canada; 5 Women’s College Research Institute, Women’s College Hospital, Toronto, Canada

## Abstract

**Background:**

Queer populations (sexual and gender diverse people, including two-spirit, lesbian, gay, bisexual, trans, and queer) in Canada face significant health disparities, largely driven by stigma related to sexual and gender identities. These inequities are associated with adverse health outcomes. Although a growing body of literature examines stigma reduction, interventions often focus on specific types of queer stigma rather than considering the broader, intersectional experiences of queer people. This scoping review aims to critically explore the current state of queer stigma across the life course in Canada, with an emphasis on understanding their impact, successes, and gaps.

**Methods:**

This review will include studies that address interventions aimed at reducing queer-related stigma in Canada. Eligible studies must focus on queer populations and measure outcomes related to stigma reduction. Studies of any design (qualitative, quantitative, mixed methods), published in English or French, and from any year will be considered. A systematic search will be conducted across multiple databases, including Medline-R (Ovid), Embase (Ovid), APA PsycInfo (Ovid), CINAHL (EBSCO), Social Services Abstracts (ProQuest), Social Science Abstracts (ProQuest), and Social Science Citation Index (Web of Science), along with grey literature sources. Two reviewers will independently assess potential articles against the inclusion criteria through two stages: titles and abstracts as well as full-text screening. Data extraction will focus on study characteristics, intervention details, and outcomes related to stigma reduction. Findings will be reported in tables and narrative summaries, guided by the socioecological model and intersectional queer theories. Data will be analyzed to identify trends and gaps in current interventions aimed at addressing queer stigma.

**Discussion:**

Using an intersectional queer approach and the socioecological model, this review will identify gaps and strengths in queer stigma programming and interventions across the life course. The broad inclusion criteria and a rigorous approach will account for academic and grey literature, highlighting ongoing treatments, measurement tools, and resources. This extensive data collection will inform future research on queer peoples’ resource and programming needs in Canada. Findings will contribute to workshops development, training modules, and community reports for healthcare providers and community organizations serving queer communities.

**Systematic review registration:**

https://doi.org/10.6084/m9.figshare.28523621.v1.

## Introduction

The alarming health inequities experienced by two-spirit, lesbian, gay, bisexual, trans, and queer (2S/LGBTQ+; herein queer) people are a significant human rights and public health issue. Queer peoples in Canada experience higher rates of mental health issues, psychological distress, substance abuse, suicidality, physical and sexual abuse, and sexually transmitted infections compared to cisgender and heterosexual counterparts [1–8]. It is generally understood that these health disparities are both the direct and indirect result of stigma associated with queer identities [9]. For example, a recent report by the Standing Committee on Health commissioned by the Canadian House of Commons notes that the health inequities experienced by queer peoples is in part due to the stigmatizations of minoritized gender and sexual identities in Canadian society and the subsequent discrimination they experienced [7]. In an effort to promote the wellbeing of queer peoples, queer health practitioners, scholars, and institutions are increasingly calling for the design, implementation, and assessment of interventions that address stigma experienced by queer peoples [7, 10, 11]. 

A stigmatized identity is a discrediting attribute or a marker of inferiority that is socially constructed and driven through various social processes by advantaged social groups. As Parker and Aggleton argue: “Stigma plays a key role in producing and reproducing relations of power and control. It causes some groups to be devalued and others to feel that they are superior in some way” (p.16) [12]. Stigma processes comprise stereotypes, or negative connotations about a marginalized group, which lead to prejudice and discrimination [13, 14]. These processes are multifaceted, with various mechanisms that operate intrapersonally (e.g., perceived or internalized stigma), interpersonally (e.g., enacted stigma), and structurally (e.g., institutional policies) [15–17]. Herek uses the terms sexual stigma and gender stigma to refer to all facets of stigma associated with sexual identity and gender identity, respectively [9]. Recognizing that the experiences of queer peoples are diverse, different, and overlapping, the proposed review employs the term queer stigma to refer generally to all stigma directed at queer populations, including sexual and gender stigma. 

Stigma towards queer individuals in Canada has deep historical roots, stemming from colonial-era impositions of heteronormative and cisnormative values. British colonial laws criminalized homosexuality until 1969, and this legal framework has entrenched negative societal attitudes that persist today [18]. Authors have argued that the decriminalization of homosexuality in Canada is a myth, and has rather been reintroduced through other polices, including indecency laws which today are still used to criminalize queer peoples [18, 19]. Although systemic barriers and queer stigma persists, Canada has made significant policy advances to promote the inclusion and rights of queer peoples, including the “decriminalization” of homosexuality, introducing anti-discrimination protections, [20] and legalizing same-sex marriage in 2005 [21]. There is an urgent need to understand stigma-mitigating efforts in Canada to address systemic issues and promote the wellbeing of queer peoples across the life course. 

## Defining stigma

Scholars have highlighted the inconsistent and varied definitions of stigma across studies and disciplines [22, 23]. Many researchers provide no explicit definition, relying instead on dictionary definitions or related concepts like stereotyping or rejection. When explicit definitions are provided, they often vary considerably [22]. 

 The socioecological model has been widely used in stigma research as an organizing framework [24, 25]. This model accounts for how stigma manifests at different levels that impact behaviour, from the individual to the structural, and can be a useful framework to study queer stigma and design interventions to overcome it. Scholars have also underscored the intersectional nature of stigma, including how its effects are compounded for individuals with multiple stigmatized identities across the life course [26–30]. While there is a growing body of literature exploring stigma interventions for queer peoples, few studies have explored queer stigma dimensions broadly [30]. Instead, existing research on interventions has focused on specific types of queer stigma, such as sexual minority stigma, [31] stigma against queer youth, [10] and HIV stigma [29]. Stigmatized identities seldom operate alone, yet interventions for stigma have often been studied in isolation [13, 29]. 

For the purpose of the following scoping review, stigma refers to socially constructed negative beliefs, stereotypes, and discriminatory actions directed at queer identities, resulting in marginalization and devaluation by dominant social groups. It operates at multiple levels—individual, interpersonal, and structural—and encompasses the overlapping impacts of sexual and gender stigmas, which intersect with other stigmatized identities across the life course. This definition was developed from a review of the literature and through discussions with stigma experts on the research team. The subsequent research approach, search strategy, and methodology will continue to use this definition to guide the development, completion, and reporting of this scoping review. 

## Research team social location and theoretical position 

Link and Phelan [22] argue that social scientists studying stigma, despite not belonging to stigmatized groups themselves, approach their research through theoretical frameworks that lack insight from the lived experiences of the populations they examine. To this end, the research team completing the proposed scoping review comprise queer scholars (with multiple intersecting identities, including non-binary and trans persons, women, and people of color), stigma experts, queer health experts, methods (qualitative, quantitative, and mixed methods) experts, implementation science and public health interventions experts, search experts, and systematic and scoping review experts. Further, this research is being conducted in collaboration with the 2S/LGBTQ+ Health Hub. The Hub is a national platform for training, mentorship, networking, and capacity-building for diverse early career researchers, trainees, healthcare professionals and service providers, and government and community-based stakeholders. The Hub aims to tackle a major training and capacity gap in intersectional and community-informed queer health and community interventions, including stigma reduction programs and health promoting interventions with and for diverse queer communities.

Our proposed study adopts a transformative paradigm, which is an activist-oriented perspective offering a framework for addressing social justice. We examine power, privilege, voice, and cultural complexity throughout the research process [32]. The axiological assumptions are respect for communities, beneficence to promote human rights and social justice, and explicit connection of research outcomes to improve social justice. A strong understanding of the community’s history and a close relationship, or integration within the community, are essential. This paradigm aligns with the review’s objectives to interrogate injustices faced by queer peoples and promote well-being through a queer-oriented approach. 

Both queer theory and intersectionality theory are critical theories that align with a transformative paradigm and have been widely used in health research to promote the wellbeing of marginalized populations [33, 34]. Queer theory is an anti-normative approach that critiques essentialist definitions of identities as well as understandings of categories and concepts [35]. Intersectionality theory is a framework that aims to understand how multiple systems of oppression intersect and interact to affect individuals and groups [33, 36, 37]. Queer theories and intersectionality have been used together in health research offering insights into the ways that health outcomes are related to complex interactions between structures of power and oppression as well as interconnected aspects of identity and social location [38, 39]. An intersectional queer approach will elucidate how queer stigma manifest as a product of a normative visions of the world that other peoples across multiple axes of identity. This focus allows for richer understandings of inequity in our social world, while illuminating experiences of stigma across the life course as products of complex systems of power, penalty, and privilege. This theoretical approach has and will inform all aspects of the research process, including conceptualization, analysis, extraction, interpretation, and reporting. 

## Research question

Our search of the extant literature revealed a paucity of reviews exploring queer stigma interventions across the life course, and notably, no studies have focused on a Canadian context. Several reviews have explored approaches to mitigating stigma and its health impacts among sexual and gender minorities. For example, Huang et al. examined intersectionality theory integration into mental health interventions for sexual minorities, highlighting a gap in addressing intersecting forms of stigma [40]. Many interventions focus on sexuality-based stigma without considering other layers like race or gender. Layland et al. reviewed psychological and behavioural health interventions, noting that while many target internalized and anticipated stigma, there is a lack of intersectionality [30]. Stangl et al. focused on reducing intersectional stigma in HIV, emphasizing the complexity of addressing multiple intersecting stigmas [41]. While interventions improved health outcomes, reducing stigma was less successful. Fowler et al. reviewed digital mental health interventions for LGBTQIA+ individuals, emphasizing community engagement in content design [42]. Digital interventions hold promise, but more targeted approaches are needed to address specific mental health challenges within the LGBTQIA+ community. These reviews underscore the importance of intersectional frameworks and addressing stigma at multiple levels to enhance health interventions for queer people. There is, however, an urgent need to explore the successes, failures, and gaps of stigma interventions and programming in Canada through an intersectional lens. Using an interdisciplinary approach within a transformative paradigm and guided by the socioecological model as an organizing framework, the proposed scoping review will aim to critically answer the question: what is the current state of queer stigma interventions across Canada? The objectives are to:

1. Map the current state of the literature describing interventions and programs seeking to reduce stigma across the life course for queer peoples across Canada.
2. Understand the successes and failures of interventions and programs seeking to reduce stigma across the life course for queer people.

## Methods

This review will follow the scoping review framework by Arksey and O’Malley and further developed by the JBI institute [43, 44]. The findings will be reported in accordance with the Preferred Reporting Items for Systematic Review and Meta-Analysis extension for Scoping Reviews (PRISMA-ScR) and PRISMA for Searching (PRISMA-S) [45, 46]. The following protocol and completed scoping review will be reported according to the Preferred Reporting Items for Systematic Review and Meta-Analysis Protocol (PRISMA-P); see Appendix 1 I [47]. The review has been registered on FigShare (https://doi.org/10.6084/m9.figshare.28523621.v1).

### Inclusion criteria

The modified PICOS will be used to guide inclusion criteria and data extraction, to accommodate qualitative and mixed methods research [48]. In a scoping review, where the goal is to map evidence broadly, PICOS elements guide inclusion criteria without necessarily limiting studies to a single type of intervention or outcome measure. This flexibility allows for a more comprehensive exploration of how stigma reduction efforts are tailored and evaluated across various populations and life stages.

### Population

Studies must focus on queer individuals at any life stage who have experienced some form of queer related stigma. The research team will highlight intersectional queer experiences, including multiple social locations, related to stigma interventions and stigma reduction. 

### Intervention

We will include studies that describe interventions or programs targeting the reduction of queer-related stigma. (i.e., stigmas experienced by queer peoples). These interventions, which must seek to reduce stigma, may include educational programs, media campaigns, social support initiatives, policy interventions, community-based programs, or therapeutic approaches, and digital interventions. The socioecological model will organize findings according to whether interventions directly target individuals, communities, or broader societal levels.

### Comparison

Scoping reviews often do not require a strict comparator. However, if comparisons are made within studies, they may include no intervention control groups, standard care of alternative interventions aimed at stigma reduction, or different approaches/delivery methods for reducing stigma. 

### Outcome

Studies should measure at least one outcome related to stigma reduction. Primary outcomes may include reductions in a form of queer-related stigma (e.g., internalized, anticipated, enacted). Secondary outcomes might involve improvements in strength-based factors, mental health, social support, knowledge, attitudes, and overall well-being.

### Study design

There will be no restrictions based on study design, as this scoping review aims to map the breadth of literature on queer stigma interventions across the life course. All relevant empirical studies (qualitative, quantitative, or mixed methods) and grey literature that provide evidence about queer stigma interventions and their impact will be included. Conceptual or theoretical papers will also be considered if they contribute to understanding the interventions' theoretical underpinnings. Reviews and editorials are excluded. 

### Geographic location

Only studies conducted in Canada, or those where Canadian data can be extracted, will be included.

### Language

 Only publications in English or French will be included.

### Search Strategy

Two members of the research team who are social science librarians (JL, JC) have designed the database search strategy in consultation with the rest of the team. The search comprises three components: stigma, queer identities, and Canada. Interventions will be identified through the screening process. A primary search was designed in Medline-R (Ovid) using both textwords and subject headings drawing on published search filters [49–51]. This search has been shared with a librarian colleague external to the team to peer review using the PRESS framework [52]. Once revisions are made, the search will be translated to the remaining databases: Embase (Ovid), APA PsycInfo (Ovid), CINAHL (EBSCO), Social Services Abstracts (ProQuest), Social Science Abstracts (ProQuest), and Social Science Citation Index (Web of Science). All databases will be searched from inception with no limits or additional filters. Search results will be downloaded on a single day and uploaded to Rayyan.AI for deduplication. The primary Medline search strategy is available in Appendix II. Supplementary search strategies will include citation searching, grey literature searching, and a journal hand search. 

### Study selection

The study selection process will be performed in two stages in Rayyan.AI, which is an AI-powered systematic review software designed to assist researchers in screening and selecting studies for inclusion in systematic reviews and scoping reviews [53]. The software uses active learning, which means it continuously learns from reviewer decisions and improves its accuracy in suggesting relevant studies. This tool helps to reduce the time and effort required for manual screening while maintaining high levels of reliability. Rayyan.AI will not be used to screen on behalf of the research team, instead, the advanced filtering and highlighting feature will be used to streamline the screening process. Following a pilot test, titles and abstract screening will be independently conducted by two members of the research team (JS and another reviewer) [53]. This will be followed by a full-text review by the same reviewers. Any conflicts at either stage will be resolved through discussion or by a third reviewer (DG). The final decision for inclusion will be based on the abovementioned criteria. 

### Data extraction

All selected studies will be documents and key characteristics will be recorded. Data extraction will be completed in Elicit.AI by one reviewer (JS) and peer reviewed by another team member [54]. Elicit.AI, an AI-powered tool, supports researchers in systematic and scoping reviews, particularly with data extraction and synthesis. The program automates and streamlines data extraction, collecting information such as intervention types, outcomes, and study design, from studies. It analyzes large volumes of studies and organizes extracted data into structured tables for further analysis and synthesis. This significantly reduces manual data extraction time, especially in large reviews. Elicit.AI learns from researcher inputs, improving its extraction capabilities over time. Researchers can train the AI by manually labeling data points, and it will automatically extract similar information from other studies. Additionally, Elicit.AI generates tables summarizing extracted data, making it easier to visualize and compare key study characteristics. This feature aids in organizing and presenting data for meta-analysis or narrative synthesis, enhancing the review process’s efficiency. All data extracted by Elicit.AI will be independently confirmed and reviewed by at least one member of the research team. The data extracted will include study characteristics (author, year, etc.), population (location, sample, demographic characteristics, etc.), intervention characteristics (type, effectiveness, etc.), outcomes measured (including measurement tools), socioecological level, theoretical underpinnings of intervention, and key findings related to the review objective. A draft data extraction tool is provided (Appendix III), which may be modified during data extraction as needed. Details of changes will be documented in the final publication. If needed, authors of articles will be contacted for missing or additional data. 

### Data analysis and presentation

Articles will not be assessed for quality as is normal for scoping reviews. Extracted data will be analyzed and synthesized to align with the objectives of the scoping review. We will present the findings in two tables. Table 1 will describe key study characteristics (e.g., author, year of publication, geographic location within Canada, and study design). Table 2 will categorize studies based on primary outcome measures related to queer stigma reduction, organized according to the socioecological model, and will describe secondary outcomes such as community support, mental health, and well-being. We will also describe the type of intervention (e.g., community-based, policy-oriented, educational) and any intersectional considerations (e.g., gender identity, age, race, sexual orientation). A narrative summary will be provided alongside the tabulated results to explain how the findings contribute to the review's objectives and address the research questions. The structure for presenting the results may be refined as the review progresses. Where possible, a visual representation (i.e., “mapping”) of the data will accompany findings. This may include showing distribution of interventions and studies across Canada, across socio-ecological levels, and/or social locations. Throughout the scoping review process, the research team will leverage the varying expertise of the scholars associated with this project to inform the direction and outcomes of the research.

## Discussion

To improve resources, supports, and interventions for queer people related to stigma and wellbeing, the landscape of current programming needs to be better elucidated. The proposed scoping review seeks to map the current state of stigma interventions for queer people across the life course in Canada. By using an intersectional queer approach and the socioecological model, this research will uncover gaps and strengths of current stigma interventions across Canada, aiming to identify key trends, effectiveness, and areas for improvement. The broad inclusion criteria and rigorous approach will allow us to account for current and past interventions documented in the academic and grey literature, highlighting successful existing treatments, measurement tools, and resources at specific socioecological levels and for particular stigmas. This extensive data collection will further inform future research studies seeking to understand the resource and programming needs of queer peoples in Canada. While there will not be any patient and public involvement in the completion of this scoping review, findings will be disseminated to trainees, community members, and researchers within the 2S/LGBTQ+ Health Hub; the findings will further contribute to the development of workshops, training modules, and community reports to be made available to health care providers and community organization staff serving queer communities.

## Limitations

For pragmatic reasons, this scoping review has been conceptualized as a review of stigma interventions for queer peoples. We have purposefully excluded search terms focusing on broader stigmas that may be overwhelmingly experienced by said population, such as HIV/AIDS stigma. Similarly, we have excluded search terms focusing on strength-based factors, including community connectedness and resilience. Studies that explore these topics will be included in the review provided they include queer populations and stigma interventions, but we recognize that this approach my omit relevant literature focusing on general populations or strength-based interventions. 

## Data Availability

Not applicable.

## Appendix I

**Table 1:** PRISMA-P (Preferred Reporting Items for Systematic review and Meta-Analysis Protocols) 487 2015 checklist: recommended items to address in a systematic review protocol*

**Section and topic**	**Item No**	**Checklist item**	**Completed (Page No.)**
**ADMINISTRATIVE INFORMATION**			
Title:			
Identification	1a	Identify the report as a protocol of a systematic review	Y (P1)
Update	1b	If the protocol is for an update of a previous systematic review, identify as such	NA
Registration	2	If registered, provide the name of the registry (such as PROSPERO) and registration number	Y (P9)
Authors:			
Contact	3a	Provide name, institutional affiliation, e-mail address of all protocol authors; provide physical mailing address of corresponding author	Y (P1)
Contributions	3b	Describe contributions of protocol authors and identify the guarantor of the review	Y (P15)
Amendments	4	If the protocol represents an amendment of a previously completed or published protocol, identify as such and list changes; otherwise, state plan for documenting important protocol amendments	NA
Support:			
Sources	5a	Indicate sources of financial or other support for the review	Y (P15)
Sponsor	5b	Provide name for the review funder and/or sponsor	Y (P15)
Role of sponsor or funder	5c	Describe roles of funder(s), sponsor(s), and/or institution(s), if any, in developing the protocol	Y (P15)
**INTRODUCTION**			
Rationale	6	Describe the rationale for the review in the context of what is already known	Y (P8)
Objectives	7	Provide an explicit statement of the question(s) the review will address with reference to participants, interventions, comparators, and outcomes (PICO)	Y (P8-10)
**METHODS**			
Eligibility criteria	8	Specify the study characteristics (such as PICO, study design, setting, time frame) and report characteristics (such as years considered, language, publication status) to be used as criteria for eligibility for the review	Y (P9-10)
Information sources	9	Describe all intended information sources (such as electronic databases, contact with study authors, trial registers or other grey literature sources) with planned dates of coverage	Y (P11)
Search strategy	10	Present draft of search strategy to be used for at least one electronic database, including planned limits, such that it could be repeated	Y (Appendix 2)
Study records:			
Data management	11a	Describe the mechanism(s) that will be used to manage records and data throughout the review	Y (P11-12)
Selection process	11b	State the process that will be used for selecting studies (such as two independent reviewers) through each phase of the review (that is, screening, eligibility and inclusion in meta-analysis)	Y (P11-12)
Data collection process	11c	Describe planned method of extracting data from reports (such as piloting forms, done independently, in duplicate), any processes for obtaining and confirming data from investigators	Y (P11-12)
Data items	12	List and define all variables for which data will be sought (such as PICO items, funding sources), any pre-planned data assumptions and simplifications	Y (P12, Appendix 3)
Outcomes and prioritization	13	List and define all outcomes for which data will be sought, including prioritization of main and additional outcomes, with rationale	Y (P12, Appendix)
Risk of bias in individual studies	14	Describe anticipated methods for assessing risk of bias of individual studies, including whether this will be done at the outcome or study level, or both; state how this information will be used in data synthesis	NA
Data synthesis	15a	Describe criteria under which study data will be quantitatively synthesized	NA
	15b	If data are appropriate for quantitative synthesis, describe planned summary measures, methods of handling data and methods of combining data from studies, including any planned exploration of consistency (such as *I*^2^, Kendall’s *τ*)	Y (P13)
	15c	Describe any proposed additional analyses (such as sensitivity or subgroup analyses, meta-regression)	NA
	15d	If quantitative synthesis is not appropriate, describe the type of summary planned	Y (P13)
Meta-bias(es)	16	Specify any planned assessment of meta-bias(es) (such as publication bias across studies, selective reporting within studies)	NA
Confidence in cumulative evidence	17	Describe how the strength of the body of evidence will be assessed (such as GRADE)	NA

## Appendix

**Table 2:** Ovid MEDLINE(R) ALL <1946 to January 22, 2025>

**#**	**Query**
1	Social stigma/
2	intoleran*.tw,kf.
3	stigma*.tw,kf.
4	homophobia/
5	biphobi*.tw,kf.
6	cisgenderis?.tw,kf.
7	cissexis?.tw,kf.
8	heteronormativ*.tw,kf.
9	heterosexis*.tw,kf.
10	homonegativ*.tw,kf.
11	homophobi*.tw,kf.
12	queerphobi*.tw,kf.
13	transphobi?.tw,kf.
14	Fear/
15	afraid.tw,kf.
16	fear*.tw,kf.
17	prejudice/
18	prejudic*.tw,kf.
19	Blame/
20	blame*.tw,kf.
21	blaming.tw,kf.
22	attitud*.tw,kf.
23	bias*.tw,kf.
24	Stereotyping/
25	stereotyp*.tw,kf.
26	Microaggression/
27	Social Discrimination/
28	disadvantage?.tw,kf.
29	discriminat*.tw,kf.
30	microaggress*.tw,kf.
31	oppress*.tw,kf.
32	privilege?.tw,kf.
33	underprivilege?.tw,kf.
34	Social Marginalization/
35	excluded.tw,kf.
36	exclusion*.tw,kf.
37	hegemon*.tw,kf.
38	inequalit*.tw,kf.
39	inequit*.tw,kf.
40	marginal*.tw,kf.
41	minority stress*.tw,kf.
42	ostraci*.tw,kf.
43	othered.tw,kf.
44	othering.tw,kf.
45	persecut*.tw,kf.
46	systemic violen*.tw,kf.
47	or/1-46 [stigma]
48	exp canada/
49	canad*.tw,kf,in.
50	(british columbia or colombie britannique or northwest territories or nouveau brunswick or nouvelle ecosse or nova scotia or prince edward island or ("new brunswick"not"new jersey") or alberta* or inuvialuit or labrador or manitoba* or newfoundland or nunavik or nunavut or nwt or ontario or quebec or saskatchewan or yukon).tw,kf,in.
51	(abbotsford or airdrie or ajax or aurora or barrie or belleville or blainville or brampton or brantford or brossard or burlington or burnaby or caledon or calgary or cape breton or chatham kent or chilliwack or clarington or coquitlam or drummondville or edmonton or fredericton or fort mcmurray or gatineau or granby or grande prairie or sudbury or guelph or halton hills or iqaluit or inuvik or kamloops or kawartha lakes or kelowna or kingston or kitchener or langley or laval or lethbridge or levis or longueuil or maple ridge or markham or medicine hat or milton or mirabel or mississauga or moncton or montreal or nanaimo or new westminster or newmarket or niagara falls or norfolk county or north bay or north vancouver or oakville or oshawa or ottawa or peterborough or pickering or port coquitlam or prince george or quebec city or red deer or regina or repentigny or richmond or richmond hill or saanich or saguenay or saint john or saint-hyacinthe or saint-jean-sur-richelieu or saint-jerome or sarnia or saskatoon or sault ste marie or sherbrooke or st albert or st catharines or st john's or strathcona county or surrey or terrebonne or thunder bay or toronto or trois-rivieres or vancouver or vaughan or whitehorse or winnipeg or wood buffalo or yellowknife or ((cambridge or (halifax or hamilton or london or victoria or waterloo or welland or whitby or windsor)) not (uk or britain or united kingdom or england or australia))).tw,kf.
52	or/48-51 [Canada]
53	Homosexuality, Male/
54	androphili*.tw,kf.
55	gay.tw,kf.
56	gays.tw,kf.
57	gbmsm*.tw,kf.
58	men who have sex with men.tw,kf.
59	mlm.tw,kf.
60	msm.tw,kf.
61	Homosexuality, Female/
62	gyn?phili*.tw,kf.
63	gyn?sexual*.tw,kf.
64	lesbi*.tw,kf.
65	sapphic.tw,kf.
66	wlw.tw,kf.
67	women loving women.tw,kf.
68	women who have sex with women.tw,kf.
69	wsw.tw,kf.
70	Bisexuality/
71	bi.tw,kf.
72	bicurious*.tw,kf.
73	bisexu*.tw,kf.
74	heteroflexible*.tw,kf.
75	panromantic*.tw,kf.
76	pansexual*.tw,kf.
77	polysexual*.tw,kf.
78	sexually fluid*.tw,kf.
79	Transsexualism/
80	"gender dysphoria"/
81	health services for transgender persons/
82	transgender persons/
83	sex reassignment surgery/
84	sex reassignment procedures/
85	afab.tw,kf.
86	amab.tw,kf.
87	cross sex*.tw,kf.
88	crosssex*.tw,kf.
89	crossgender*.tw,kf.
90	cross gender*.tw,kf.
91	csh.tw,kf.
92	csht.tw,kf.
93	"female-to-male".tw,kf.
94	f2m.tw,kf.
95	gaht.tw,kf.
96	m2f.tw,kf.
97	"male-to-female".tw,kf.
98	transfol*.tw,kf.
99	transperson*.tw,kf.
100	transsexual*.tw,kf.
101	transgender*.tw,kf.
102	transpeople*.tw,kf.
103	tgnc*.tw,kf.
104	ytw.tw,kf.
105	(trans adj2 (people or individual or individuals or person or persons or sexual* or man or men or male or female or youth* or woman or women or population* or gender or folk)).tw,kf.
106	((gender* or sex or sexual) adj2 (affirm* or change or dysphoria or reversal or reassign* or transform* or transition* or minorit* or binary or incongruen* or divers* or discord*)).tw,kf.
107	Transvestism/
108	cross dress*.tw,kf.
109	crossdress*.tw,kf.
110	transvest*.tw,kf.
111	"Intersex Persons"/
112	intersex*.tw,kf.
113	inter sex*.tw,kf.
114	Gender-Nonconforming Persons/
115	agender*.tw,kf.
116	bi-gender*.tw,kf.
117	bigender*.tw,kf.
118	enby.tw,kf.
119	enbies.tw,kf.
120	gender fluid*.tw,kf.
121	gender non-conforming.tw,kf.
122	gender-expansive.tw,kf.
123	genderqueer*.tw,kf.
124	inter gender*.tw,kf.
125	intergender*.tw,kf.
126	neutrois.tw,kf.
127	non-cisgender*.tw,kf.
128	non-gender*.tw,kf.
129	nonbinar*.tw,kf.
130	pangender*.tw,kf.
131	poly-gender*.tw,kf.
132	polygender*.tw,kf.
133	third gender*.tw,kf.
134	polyamor*.tw,kf.
135	poly amor*.tw,kf.
136	2 spirit*.tw,kf.
137	2s.tw,kf.
138	2spirit*.tw,kf.
139	blaq.tw,kf.
140	blaqueer*.tw,kf.
141	indigiqueer*.tw,kf.
142	qpoc.tw,kf.
143	qtpoc.tw,kf.
144	sgl.tw,kf.
145	two spirit*.tw,kf.
146	twospirit*.tw,kf.
147	asexual*.tw,kf.
148	ace.tw,kf.
149	graysexual*.tw,kf.
150	gray sexual*.tw,kf.
151	demisexual*.tw,kf.
152	(aroman* or a-roman*).tw,kf.
153	Homosexuality/
154	exp"Sexual and Gender Minorities"/
155	GLB*.tw,kf.
156	GLBT*.tw,kf.
157	GLBQ*.tw,kf.
158	GLBs*.tw,kf.
159	homosexual*.tw,kf.
160	LGB*.tw,kf.
161	LGBQ*.tw,kf.
162	LGBS*.tw,kf.
163	LGBT*.tw,kf.
164	queer*.tw,kf.
165	same gender.tw,kf.
166	same sex.tw,kf.
167	sexual minorit*.tw,kf.
168	or/53-167 [All Queer terms]
169	47 and 52 and 168

## Appendix III

**Table 3:** Draft data extraction table.

Author	
Title	
Year	
Journal	
Funding Source	
Location in Canada	
Article type	
Methods	
Study Design	
Population	
Intersectionality considerations	
Sample Size	
Intervention characteristics (type, duration, mode of administration, setting, feasibility/acceptability, intervention adaptation, follow-up retention etc.)	
Primary outcomes (stigma related)	
Socioecological level(s)	
Secondary outcomes (strength-based, community, wellbeing etc.)	
Theoretical underpinnings	
Impact of intervention (e.g., effect sizes, qualitative description)	
Key findings	
Implications	
Limitations	
Ethical Considerations	
